# Cardiovascular risk prediction in healthy older people

**DOI:** 10.1007/s11357-021-00486-z

**Published:** 2021-11-11

**Authors:** Johannes T. Neumann, Le T. P. Thao, Emily Callander, Enayet Chowdhury, Jeff D. Williamson, Mark R. Nelson, Geoffrey Donnan, Robyn L. Woods, Christopher M. Reid, Katrina K. Poppe, Rod Jackson, Andrew M. Tonkin, John J. McNeil

**Affiliations:** 1 Department of Epidemiology and Preventive Medicine, School of Public Health and Preventive Medicine, Monash University, 99 Commercial Road, Melbourne, Victoria 3004 Australia; 2 Department of Cardiology, University Heart & Vascular Centre, Hamburg, Germany; 3 German Centre for Cardiovascular Research (DZHK), Partner Site Hamburg/Kiel/Lübeck, Hamburg, Germany; 4 School of Public Health, Curtin University, Perth, WA Australia; 5 Sticht Centre On Aging and Alzheimer’s Prevention, Section On Gerontology and Geriatric Medicine, Department of Internal Medicine, Wake Forest School of Medicine, Winston-Salem, NC USA; 6 Menzies Institute for Medical Research, University of Tasmania, Hobart, Australia; 7 Melbourne Brain Centre, Royal Melbourne Hospital, University of Melbourne, Parkville, VIC Australia; 8 Epidemiology & Biostatistics, University of Auckland, Auckland, New Zealand; 9 Department of Medicine, University of Auckland, Auckland, New Zealand

**Keywords:** Risk prediction, Major adverse cardiovascular event, MACE, Elderly, Model, Risk factors

## Abstract

Identification of individuals with increased risk of major adverse cardiovascular events (MACE) is important. However, algorithms specific to the elderly are lacking. Data were analysed from a randomised trial involving 18,548 participants ≥ 70 years old (mean age 75.4 years), without prior cardiovascular disease events, dementia or physical disability. MACE included coronary heart disease death, fatal or nonfatal ischaemic stroke or myocardial infarction. Potential predictors tested were based on prior evidence and using a machine-learning approach. Cox regression analyses were used to calculate 5-year predicted risk, and discrimination evaluated from receiver operating characteristic curves. Calibration was also assessed, and the findings internally validated using bootstrapping. External validation was performed in 25,138 healthy, elderly individuals in the primary care environment. During median follow-up of 4.7 years, 594 MACE occurred. Predictors in the final model included age, sex, smoking, systolic blood pressure, high-density lipoprotein cholesterol (HDL-c), non-HDL-c, serum creatinine, diabetes and intake of antihypertensive agents. With variable selection based on machine-learning, age, sex and creatinine were the most important predictors. The final model resulted in an area under the curve (AUC) of 68.1 (95% confidence intervals 65.9; 70.4). The model had an AUC of 67.5 in internal and 64.2 in external validation. The model rank-ordered risk well but underestimated absolute risk in the external validation cohort. A model predicting incident MACE in healthy, elderly individuals includes well-recognised, potentially reversible risk factors and notably, renal function. Calibration would be necessary when used in other populations.

**Supplementary Information:**

The online version contains supplementary material available at 10.1007/s11357-021-00486-z.

## Introduction

Atherothrombotic cardiovascular disease (CVD) is a very important non-communicable disease worldwide and associated with high morbidity and mortality [1–4]. The WHO has declared CVD as one of the priority diseases within their action plan, aiming to reduce CVD-related mortality by 25% by 2025 [5]. A key requirement to achieve this goal is to improve the prediction of incident CVD events. This requires identification of individuals at highest risk of major adverse cardiovascular events (MACE) to target effective interventions.

Modifiable and non-modifiable risk factors have been incorporated in algorithms aimed to estimate an individual’s risk of future cardiovascular events [4, 6–9]. These algorithms include traditional risk factors, and estimate the future risk of fatal or nonfatal events, over a period of 5 or 10 years. An important limitation of most risk scores is that the population cohort data from which they were derived have included individuals whose median age is typically less than 70 years. However, with the ageing of high-income countries, most cardiovascular events now occur in the elderly, beyond the range of most existing equations.

As an example, the European Society of Cardiology (ESC) Systematic COronary Risk Evaluation 2 (SCORE2) is limited to the age range from 40 to 65 years and the median age of the recently published LIFE-CVD cohorts was approximately 60 years [9, 10]. Several studies have demonstrated that SCORE, Framingham and other similar algorithms are less effective in predicting cardiovascular events in the elderly [11, 12]. Thus, recent ESC guidelines have not recommended their use in individuals over 70 years, as the risk for cardiovascular events might be overestimated due to competing causes of death [4, 13].

CVD risk prediction models developed specifically for use in the elderly also have important limitations. The recently published SCORE2 Older Persons (OP) equation estimates future risk of incident CVD events [14]. However, it is only based on established risk factors included in the original SCORE project and in addition, high-density lipoprotein cholesterol (HDL-c) and diabetes, while predictors such as renal function or comorbidities were not considered. Prediction algorithms developed from the PROSPER and ARIC studies were based on smaller and less contemporary cohorts and had a shorter duration of follow-up [15, 16]. More recent findings also suggest that traditional risk factors for CVD might be weaker predictors of future risk in the elderly while the strength of other factors such as chronic kidney disease increases with ageing [11]. Therefore, improved prediction of CVD events in the elderly represents an unmet clinical need.

Given these limitations, we aimed to develop a risk prediction model for incident MACE from subjects enrolled in a large clinical trial in initially healthy, elderly individuals and to validate the model in a large primary care dataset.

## Methods

### Derivation dataset

For the derivation of the risk model, data from the ASPirin in Reducing Events in the Elderly (ASPREE) study was analysed. Details of the ASPREE study have been reported previously [17–19]. Briefly, it was a randomised, placebo-controlled trial investigating the effect of low-dose aspirin on disability-free survival in healthy, elderly people. Cardiovascular events were amongst the prespecified secondary endpoints. In total, 19,114 community-dwelling individuals aged ≥ 70 years (≥ 65 for US minorities), without prior CVD events, dementia or physical disability were recruited in Australia and the USA. Specific exclusion criteria are reported in the Supplementary Material. For the present analyses, we also excluded all participants aged < 70 years, as they comprised US minorities. All participants provided written informed consent. The study was approved by local Ethics Committees and is registered on clinicaltrials.gov (NCT01038583).

### Potential predictors investigated

All baseline variables were investigated as potential predictors and collected as part of the standardised recruitment process. The initial selection of such predictors was based on prior research and risk models, and included age, sex, smoking history, systolic and diastolic blood pressure, current use of antihypertensive agents, HDL-c, non-HDL-c, serum creatinine, diabetes, body mass index (BMI), haemoglobin, family history of myocardial infarction (MI) and an area-based measure of socioeconomic status (the Index of Relative Socio-economic Advantage and Disadvantage (IRSAD) score). Details concerning these predictors are provided in the Supplementary Material.

### Endpoint

The primary endpoint for our analyses was a composite of incident MACE. This included coronary heart disease (CHD) death, nonfatal MI and fatal or nonfatal ischaemic stroke. Causes of CHD death included MI, sudden or rapid cardiac death and other CHD death, but not death from heart failure which was not attributable to CHD. All events were adjudicated by expert committees blinded to treatment allocation, as described previously [18].

### External validation dataset

The PREDICT cohort study, which has been described before in detail, was used as an external validation dataset [8]. Briefly, PREDICT is an ongoing cohort study in New Zealand, which automatically enrols participants without prior events having absolute CVD risk assessment in primary care. Using linkage to national hospitalisation and mortality databases based on International Classification of Disease (ICD) codes, incident events are captured. For the present analyses, we only considered PREDICT participants aged 70–79 years at baseline, of self-reported European ethnicity and without CVD. To replicate the ASPREE exclusion criteria, we further excluded PREDICT participants having certain ICD codes, particularly related to cancer at baseline or the prior 5 years (Supplementary Material). The MACE endpoint in the validation dataset was based on ICD codes and included MI, ischaemic stroke and death from CHD (Table S1).

### Statistical analyses

Cox proportional hazard regression models were used to calculate the 5-year predicted risk of MACE. Participants who died for reasons other than MACE were censored at the time of death. To allow estimation of predicted risks in the external data, baseline survival probability at 5 years was obtained by computing the survival function of a reference participant. A reference participant was a subject whose all covariates were equal to zero.

As the amount of missing data for most variables was low (< 5%), and it was reasonable to be considered as missing at random, a complete case analysis was used to develop prediction models [20]. However, the IRSAD score was missing in all US participants and was therefore only considered in sensitivity analyses.

Prior to variable selection, we tested for potential non-linear relationships of continuous variables and the outcome by modelling them as restricted cubic spline functions with two degrees of freedom. From the initial selected predictors, a variable was incorporated in the final model if it showed a strong association with the outcome in the data (*p* < 0.05 in univariable analyses) or because it was an established CVD predictor in prior research.

To validate the variable selection, we additionally performed a machine-learning variable selection using the least absolute shrinkage and selection operator (lasso) in combination with bootstrapping. Sex-specific models were examined as an exploratory analysis.

Regarding model performance, the cumulative/dynamic receiver operating characteristic curves and the area under the curve (AUC) for 5-year risk were used to evaluate discrimination with each of the selected predictors and the final model. To assess the agreement between predicted and observed risks at 5 years, we used calibration plots [21]. The model performances were corrected for overfitting by internal bootstrap validation. Specifically, the selected predictors were fitted to each bootstrap sample to obtain bootstrap final models. The optimism due to overfitting was then obtained by comparing the performance of the bootstrap final models on the original dataset and bootstrap datasets. We calculated the bias-corrected model performance by subtracting the estimated optimism from the original model performance. The derived model was then applied in the validation dataset, and AUC and calibration evaluated. Finally, we aimed to compare the model performance with the recently published SCORE2-OP model.

All analyses were performed using R version 3.6.1 (R Foundation for Statistical Computing, Vienna, Austria, www.r-project.org).

## Results

### Baseline characteristics of the derivation dataset

The derivation dataset consisted of 18,548 participants with a mean age of 75.4 years, of whom 10,426 (56.2%) were female (Table 1). The majority were in the age categories ranging from 70 to 74 years (57.1%) and 75 to 79 years (27.1%), while only 3.9% were aged above 85 years. When comparing males and females, only minor differences were observed (Table S2). Most continuous variables in the dataset showed only low to moderate correlation (Figure S1).

**Table 1:** Baseline characteristics of the derivation dataset

	**Overall**	**70–74 years**	**75–79 years**	**80–84 years**	** ≥ 85 years**
*N* total	18,548 (100)	10,598 (57.1)	5,022 (27.1)	2,196 (11.8)	732 (3.9)
Australian participants (%)	16,703 (90.1)	9,669 (91.2)	4,431 (88.2)	1,963 (89.4)	640 (87.4)
US participants (%)	1,845 (9.9)	929 (8.8)	591 (11.8)	233 (10.6)	92 (12.6)
Age (mean (SD))	75.35 (4.39)	72.28 (1.35)	77.16 (1.43)	82.03 (1.38)	87.41 (2.10)
Female sex (%)	10,426 (56.2)	5,782 (54.6)	2,914 (58.0)	1,295 (59.0)	435 (59.4)
Current smoker (%)	656 (3.5)	436 (4.1)	159 (3.2)	45 (2.0)	16 (2.2)
Systolic blood pressure, mmHg (mean (SD))	139.35 (16.48)	138.32 (16.18)	139.92 (16.58)	141.53 (16.96)	143.62 (17.21)
BMI, kg/m^2^ (mean (SD))	28.02 (4.66)	28.29 (4.70)	27.99 (4.72)	27.30 (4.28)	26.37 (4.14)
Haemoglobin, g/dL (mean (SD))	14.18 (1.21)	14.28 (1.21)	14.11 (1.19)	13.97 (1.19)	13.76 (1.19)
HDL-c, mmol/L (mean (SD))	1.59 (0.46)	1.57 (0.46)	1.59 (0.45)	1.61 (0.48)	1.65 (0.46)
Non-HDL-c, mmol/L (mean (SD))	3.67 (0.94)	3.71 (0.94)	3.64 (0.93)	3.59 (0.95)	3.54 (0.96)
Diabetes (%)	1,900 (10.2)	1,025 (9.7)	554 (11.0)	236 (10.7)	86 (11.7)
Serum creatinine, mg/dL (mean (SD))	0.91 (0.22)	0.89 (0.21)	0.91 (0.22)	0.94 (0.24)	0.96 (0.26)
Family history of MI (%)	473 (2.6)	261 (2.5)	140 (2.8)	58 (2.6)	14 (1.9)
IRSAD score (mean (SD))	1,003 (69)	1,004 (70)	1,001 (68)	1,002 (67)	1,007 (69)
Intake of antihypertensive agents (%)	9,712 (52.4)	5,262 (49.7)	2,741 (54.6)	1,255 (57.1)	454 (62.0)

For all continuous variables, the mean or median and the standard deviation (SD) or interquartile range are reported. For binary variables, absolute and relative frequencies are provided. Missing values for continuous variables were 1,893 for IRSAD score, 463 for creatinine, 446 for non-HDL-c, 444 for HDL-c, 87 for BMI and 2 for haemoglobin. Abbreviations: *US*, United States; *SD*, standard deviation; *BMI*, body mass index; *HDL-c*, high-density lipoprotein cholesterol; *MI*, myocardial infarction; *IRSAD*, Index of Relative Socio-economic Advantage and Disadvantage

### Development of the prediction model

During the median follow-up time of 4.7 years (interquartile range 3.6–5.7), 594 incident MACE were recorded (Table S3). In univariable regression analyses, diastolic blood pressure, BMI and family history of MI were not significant predictors for incident MACE (Table S4, Figure S2).

When considering all predictors in one model, only age, sex, current smoking, systolic blood pressure, non-HDL-c, HDL-c, serum creatinine and intake of antihypertensive agents were significant predictors (Table S5). Thus, diastolic blood pressure, BMI, haemoglobin and family history of MI were not considered for the final model. However, diabetes was forced into the model, because it is an established cardiovascular risk factor.

**Fig. 1:**
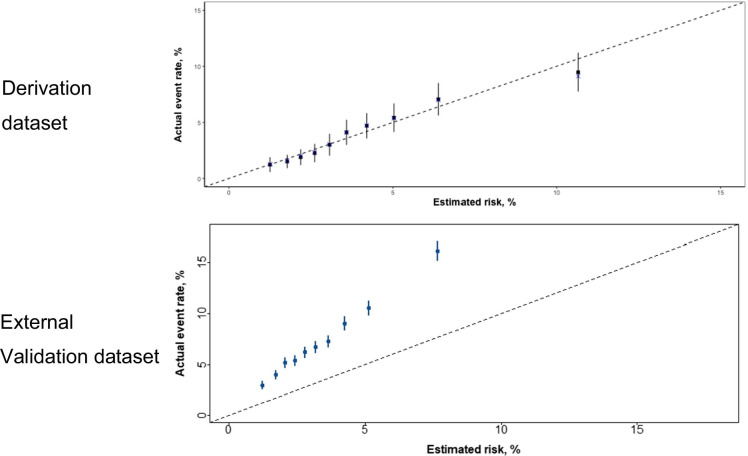
Calibration plot of the prediction model. The black dots compare the observed and the predicted MACE probability together with the 95% confidence intervals. The blue crosses for the validation dataset represent biased-corrected predicted risk based on 200 bootstrap samples

The final algorithm was developed from 17,742 individuals (Table 2). Increasing age, being a current smoker or having diabetes, taking antihypertensive agents, or having higher systolic blood pressure, non-HDL-c or serum creatinine increased the risk of MACE, while being female or increasing HDL-c decreased the risk of MACE (Figure S3). The final model had an AUC of 68.11 (95% confidence interval [CI] 65.86; 70.36) (Table S6). The final model demonstrated good calibration in the derivation dataset, with similar predicted and observed risks of MACE (Fig. 1). This finding was also confirmed, when evaluating calibration separately for males and females (Figure S4). The internal validation of the model showed a bias-corrected AUC of 67.52 (Table S6). Application of the SCORE2-OP model in the ASPREE population resulted in an AUC of 66.31 (95% CI 64.00; 68.61) (Table S6).

**Table 2:** Multivariable regression model for prediction of incident MACE in the derivation dataset

	**Hazard ratio**	**95% CI**	***p*-value**
Age per year	1.09	(1.07; 1.10)	< 0.001
Female sex (yes/no)	0.61	(0.50; 0.74)	< 0.001
Current smoking (yes/no)	1.95	(1.38; 2.76)	< 0.001
Diabetes (yes/no)	1.20	(0.93; 1.55)	0.16
Intake of antihypertensive agents (yes/no)	1.31	(1.11; 1.56)	0.002
Systolic blood pressure per 10 mmHg	1.06	(1.01; 1.12)	0.013
Non-HDL-c per mmol/L	1.29	(1.18; 1.41)	< 0.001
HDL-c per mmol/L	0.77	(0.62; 0.95)	0.016
Serum creatinine per 0.1 mg/dL	1.05	(1.02; 1.09)	0.005

This model is based on 17,742 individuals and 594 events. Abbreviations: *CI*, confidence interval; *HDL-c*, high-density lipoprotein cholesterol. Baseline survival function at 5 years (*S*0(5 years)) is 0.9999872. The predicted risk at 5 years is calculated using this formula: 1 − *S*0(5 years)^exp(PI), with PI = Age * 0.08161426 + Gender * − 0.49972875 + Serum Creatinine * 0.50978624 + non-HDL-c * 0.25461620 + HDL-c * − 0.26648903 + Current smoking * 0.66701137 + Systolic blood pressure * 0.006212068 + Diabetes * 0.18337034 + Intake of antihypertensive agents * 0.27318009

In an alternative approach to variable selection based on machine-learning, age, sex, serum creatinine, non-HDL-c, HDL-c and systolic blood pressure were identified as the most important predictors, with each of them being selected in more than 50% of cases (Table 3).

**Table 3:** Weighting of variable selection using least absolute shrinkage and selection operator in the derivation dataset

Predictor	%
Age	100.0%
Sex	99.5%
Serum creatinine	95.0%
Non-HDL-c	91.5%
HDL-c	77.5%
Systolic blood pressure	53.5%
Current smoking	36.5%
Intake of antihypertensive agents	23.0%
Diabetes	7.0%
Family history of MI	3.0%
BMI	2.0%
Haemoglobin	1.5%

Abbreviations: *BMI*, body mass index; *HDL-c*, high-density lipoprotein cholesterol; *MI*, myocardial infarction

### External validation

The external validation dataset consisted of 25,138 participants whose baseline characteristics were similar to the derivation dataset (Table S7). Their mean age was 73.2 years (SD 2.62), 53% were females, 5% were current smokers, 13% had diabetes, mean systolic blood pressure was 136 mmHg (SD 14.9), mean non-HDL-c concentration was 3.6 mmol/L (SD 0.99) and mean serum creatinine concentration was 0.91 mg/dL (SD 0.21).

During a median follow-up time of 6.4 years, 2,340 MACE were observed (Table S3). The final model showed an AUC of 64.16 (95% CI 62.77; 65.55, Table S6). The model effectively rank-ordered risk of MACE; however, the score clearly underestimated absolute risk in the validation cohort (Fig. 1).

### Sensitivity analyses

In sensitivity analyses, we included the IRSAD score as an indicator of socioeconomic status, but it was not shown to be an independent predictor of MACE (Table S8). When also including the IRSAD score in the lasso selection, this showed a low inclusion frequency (Table S9).

We also calculated the model for males and females separately (Table S10, Figure S3). In males, age, current smoking, systolic blood pressure, non-HDL-c and serum creatinine remained independent predictors, while HDL-c, diabetes and intake of antihypertensive agents were not. In females, age, current smoking, non-HDL-c and intake of antihypertensive agents remained independent predictors, while systolic blood pressure, HDL-c, diabetes and serum creatinine were not.

## Discussion

**Fig. 2:**
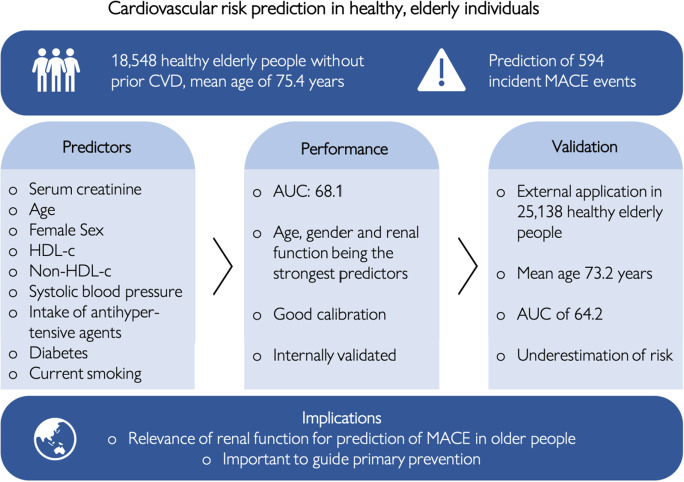
Summarising figure

In a large, contemporary population of healthy, elderly individuals in Australia and the US who volunteered for a clinical trial, we developed a model predicting incident MACE. This model includes age, sex, current smoking, systolic blood pressure, HDL-c, non-HDL-c, serum creatinine, diabetes and intake of antihypertensive agents as predictors and resulted in good discrimination and calibration in internal validation analyses (Fig. 2). Importantly, the model was externally validated in a large dataset of older people undergoing cardiovascular risk assessment in primary care in New Zealand. The model effectively ranked older individuals in order of MACE risk but significantly underestimated the absolute risk in this validation cohort.

Most established risk prediction models have excluded elderly individuals. This applies to the well-established ESC SCORE2, which is only recommended for individuals aged below 66 years [10]. The atherosclerotic CVD score is also limited to an upper age of 79 years [22] and has been shown to perform substantially worse in older than in younger individuals [12].

There are relatively few specific risk models for elderly individuals. The SCORE2 Older Persons risk model is the most recognised [14], and was validated in different external cohorts. Another risk model was based on the PROSPER study, which included 5,146 elderly individuals without CVD [16]. The PROSPER cohort was substantially different from ASPREE. It is no longer contemporary, as recruitment commenced in 1997, and it represents a high-risk population with 33% current smokers and mean systolic blood pressure very high, being 157 mmHg.

In contrast to these previous models, the ASPREE model was derived from a contemporary, healthy population with fairly well-controlled risk factors, a high degree of antihypertensive or lipid-lowering drug use, contributing to a lower CVD event rate than expected from prior data. Importantly, due to rigorous screening at baseline, we can be confident that all participants had no prior CVD events.

Although caution is needed in comparing findings from different cohorts, the discrimination of the model, with an AUC of 0.68, was higher than observed with other models in older people. The atherosclerotic CVD score resulted in an AUC of 0.62 and the original validation of the SCORE2-OP in an AUC of 0.63 [12, 14, 23]. When the SCORE2-OP model was applied in ASPREE, it resulted in an AUC of 0.66. However, the AUC was lower compared to the performance of other risk models developed in younger individuals, where the AUC usually ranged between 0.70 and 0.75 [24]. This could be explained by the greater importance of competing mortality risks in the elderly and the fact that their more frequent multimorbidity makes MACE adjudication more difficult. This older cohort also has a narrow age range and therefore more homogeneous, limiting capacity for discrimination.

The model was externally validated in the large PREDICT cohort, which consists of community-dwelling people without prior CVD events undergoing absolute CVD risk estimation. The primary care environment is ideal for validation of the model, as it is the setting in which application in clinical practice typically occurs. Importantly, we were able to mimic the ASPREE population in the validation dataset by applying ICD codes for prior CVD events, cancer diagnoses and cognitive impairment. As usually expected in validation studies, the discrimination was lower in PREDICT compared to ASPREE. Importantly, absolute CVD risk was significantly underestimated in the validation cohort. There are several possible explanations for this. These include the relatively low risk of the ASPREE population, likely contributed to by a healthy-volunteer effect. Event ascertainment was also different, as ASPREE used the strict adjudication process of a clinical trial, while PREDICT used ICD codes. Furthermore, inter-country differences in CVD risk between Australia and New Zealand may be important. Finally, there remain some differences between the derivation and validation populations, as individuals with major physical disability, atrial fibrillation or serious illness were excluded in ASPREE, but not in PREDICT. Therefore, recalibration of the ASPREE model according to the risk in the particular population in which the equation would be applied should be undertaken. This has for example been done for the ESC SCORE, distinguishing low- and high-risk countries [4].

An important difference from previous models is also the time frame for risk prediction. Most established risk models provide estimates for events over 10 years. However, a shorter time horizon of 5 years may be more appropriate in the elderly, due to their limited life expectancy and the high frequency of competing causes of death, especially cancer [15]. This shorter time frame also enables more precise modelling and presentation of the potential impact of evidence-based interventions as it approximates the duration of most CVD prevention trials. Furthermore, focus group testing of elderly has found that consumers value nearer life years more highly and discounted events further into the future [25].

In our analyses, we provide significant support for the relevance of traditional predictors in elderly individuals. That age remains the dominant predictor was expected. This was also confirmed in a recent analysis of 12 predictors using artificial intelligence which also found that age was the most important [26]. Sex, smoking, lipids, systolic blood pressure and intake of antihypertensive agents are also well-established risk factors and remain important predictors in the elderly. The persisting relevance of modifiable risk factors emphasises the role of primary prevention in the elderly. This particularly applies to elevated blood pressure, as shown in the SPRINT trial [27], as well as to elevated lipids. Here, recent data suggests not only an association of elevated lipids with MACE but also a benefit of statin treatment in elderly people [28, 29]. The reversibility of the risk of CVD conferred by elevated lipids and the benefit of statin treatment in elderly people are also being addressed in the ongoing STAREE and PREVENTABLE trials (NCT02099123, NCT04262206).

After age and sex, we found impaired renal function to be the third most frequently selected predictor in our machine-learning analysis. Although common in an elderly population, measures of impaired renal function have not been included in risk models developed in younger individuals, in whom impaired renal function is less frequent. In a recent analysis of the atherosclerotic CVD score in elderly individuals, the estimated glomerular filtration rate (eGFR) was substantially lower in elderly [12] and the PROSPER risk model included the eGFR as risk factor [16]. In our analysis, we focused on serum creatinine instead of the eGFR, as creatinine is more widely available and the adjustment for age contained in the eGFR model was less relevant.

Intriguingly, diabetes, although included in our model, was not identified as a significant predictor of MACE. This contrasts with recent analyses in elderly participants from three population-based studies, in which diabetes was a significant predictor for CHD events [12]. Only one study, the Finland Italy Netherlands Elderly study, did not show diabetes to be a significant predictor in elderly individuals [30]. This raises the question about whether the importance of diabetes as a risk factor might depend on the age at the time of diagnosis and therefore duration of exposure to risk. It is also noteworthy that a high proportion of the ASPREE participants were receiving treatment with statins and blood pressure lowering agents, particularly angiotensin converting enzyme inhibitors which may have impacted on the risk conferred by diabetes (Table S11). Finally, some diabetic patients would have been identified at initial study recruitment as part of screening.

The current burden of CVD in communities of advanced age is well established, with numerous studies documenting the increased requirement for hospital and community-based health services, as well as increased use of residential care facilities after CVD events [31]. Those who experience a CVD event are often unable to continue their usual activities, with many of those affected becoming increasingly reliant on government support [32, 33]. The identification of individuals at risk of MACE and successful early preventative intervention can thus have society-wide benefits. Given the ageing of populations globally [34], and thus the increasing numbers of individuals at high risk of CVD events, avoidance of the costs associated with CVD is a priority for governments. The routine use of a specific MACE prediction model offers an important way of minimising these costs.

This present investigation not only has certain strengths but also limitations. The major strength is the contemporary, high-quality dataset, which was used for the analyses. ASPREE, being a randomised controlled trial with a prespecified secondary outcome focused on CVD, provides systematic measurement of risk factors at baseline in a unique dataset of elderly individuals, free of CVD events at baseline. The robust ascertainment and adjudication process of all MACE outcomes also represents an important strength compared to other population-based studies. Another strength is clear definition of the population to whom these data are generalisable. Finally, we were able to apply the risk model in the family practice environment in the large PREDICT cohort, which shares important similarities with ASPREE. This resulted in a total of 43,686 elderly individuals available for our analyses.

Amongst the limitations, the data were obtained from a large clinical trial rather than a random sample from the general population. However, recruitment to a clinical trial allows formal exclusion of those with prior CVD events. In addition, the validation in the community-based PREDICT cohort showed similar findings particular in rank-ordering risk, albeit underestimating absolute risk. Amongst other limitations, the number of events in the derivation dataset was lower than would be expected in a general population. This is very likely explained by the healthy cohort effect resulting in better health status of the participants at baseline. Finally, our analyses were derived from a mainly Caucasian population and thus care may be needed in applying the model to other populations.

In conclusion, the ASPREE population reflects the increasingly large group of Caucasian individuals aged 70 years and over without CVD events, in general good health and who would be considered for primary prevention strategies [15]. In our analyses, we confirmed the importance of age, sex, systolic blood pressure, smoking and lipids for prediction of future MACE in a population of healthy, elderly individuals. We also identified impaired renal function as an important predictor, which has not been recognised in prior CVD risk models.

## Supplementary Information

Below is the link to the electronic supplementary material.

Supplementary file1 (DOCX 645 KB)
