# Supplementary material for: Cardiovascular risk prediction in healthy older people
Source: GeroScience. 2021 Nov 11;44(1):403–13. doi: 10.1007/s11357-021-00486-z (PMC8810999; doi:10.1007/s11357-021-00486-z)
Supplement: Supplementary file 1 — Supplementary file1 (DOCX 645 KB) [file 11357_2021_486_MOESM1_ESM.docx]

**Supplementary Material to**

Cardiovascular risk prediction in healthy older people

*J.T. Neumann et al.*

**Content**

1. Supplementary Material page 2
2. Supplementary Results page 4
3. Supplementary Tables page 5
4. Supplementary Figures page 15

**Supplementary Material**

ASPREE exclusion criteria

Individuals were specifically excluded if they had a history of a diagnosed CVD event defined as previous myocardial infarction (MI), heart failure, angina pectoris, stroke, transient ischaemic attack, 50% carotid artery stenosis or previous carotid endarterectomy or stenting, coronary artery angioplasty or stenting, coronary artery bypass grafting, or abdominal aortic aneurysm. Individuals with atrial fibrillation, a high risk of bleeding or serious illness likely to cause death within the next 5 years were also excluded.

Potential predictors investigated (Derivation dataset)

Smoking was defined as current smoking. For blood pressure, the mean value from three measurements in a sitting position was used. Diabetes mellitus was defined as either self-reported, a fasting glucose ≥ 126 mg/dL or being on drug treatment for diabetes. A family history of MI was defined by either father, mother or sibling having had this prior to the age of 50 years. The prescription of antihypertensive agents was coded by using the Anatomical Therapeutic Chemical codes and included the categories C02 (antihypertensives), C03 (diuretics), C07 (beta blocking agents), C08 (calcium channel blockers), and C09 (agents acting on the renin-angiotensin system). The IRSAD was developed by the Australian Bureau of Statistics and is a continuous value, which provides an objective measure of the relative economic and social conditions of residents from a defined postcode.

External validation dataset

Using linkage to national hospitalization and mortality databases based on International Classification of Disease (ICD) codes, incident events are captured. For the present analyses we only considered PREDICT participants aged 70-79 years at baseline, of self-reported European ethnicity, and without CVD. To replicate the ASPREE exclusion criteria, we further excluded PREDICT participants having certain ICD codes, particularly related to cancer at baseline or the prior 5 years (Supplementary Material). The MACE endpoint in the validation dataset was based on ICD codes and included MI, ischemic stroke and CHD death (Table S1).

ICD codes used for exclusion in the validation dataset, if recorded at baseline or within the prior 5 years

F00 (Alzheimer disease)

F01 (Vascular dementia)

F02 (other dementia)

F03 (unspecified dementia)

C15 (Malignant neoplasm of esophagus)

C16 (Malignant neoplasm of stomach)

C22 (Malignant neoplasm of liver and intrahepatic bile ducts)

C23 (Malignant neoplasm of gallbladder)

C24 (Malignant neoplasm of other and unspecified parts of biliary tract)

C25 (Malignant neoplasm of pancreas)

C34 (Malignant neoplasm of bronchus and lung)

C64 (Malignant neoplasm of kidney)

C71 (Malignant neoplasm of brain), and C90 (Multiple myeloma).

**Supplementary Results**

Sensitivity analyses

In sensitivity analyses, we included the IRSAD score as an indicator of socioeconomic status, but it was not shown to be an independent predictor of MACE (Table S7). When also including the IRSAD score in the lasso selection, this showed a low inclusion frequency (Table S8).

We also calculated the model for males and females separately (Table S9). In males, age, current smoking, systolic blood pressure, non-HDL-c and serum creatinine remained independent predictors, while HDL-c, diabetes and intake of antihypertensive agents were not. In females, age, current smoking, non-HDL-c and intake of antihypertensive agents remained independent predictors, while systolic blood pressure, HDL-c, diabetes and serum creatinine were not (Figure S2).

**Supplementary Tables**

Table S1: ICD codes used for endpoint definition in the validation dataset

| Outcome | ICD code |
| --- | --- |
| Myocardial infarction | I21 |
| Ischemic stroke | I63 |
| CHD death | I20, I21, I22, I23, I24, I25, I46, I63, R96, R98 |

Abbreviations: CHD = coronary heart disease; ICD = International Classification of Diseases.

Table S2: Baseline characteristics for males and females in the derivation dataset

|  | Male | Female | p-value |
| --- | --- | --- | --- |
| N total | 8,122 | 10,426 |  |
| Australian participants (%) | 7,524 (92.6) | 9,179 (88.0) |  |
| US participants (%) | 598 (7.4) | 1,247 (12.0) | <0.001 |
| Age (mean (SD)) | 75.16 (4.33) | 75.51 (4.43) | <0.001 |
| Age categories (%) |  |  |  |
| 70-74 | 4,816 (59.3) | 5,782 (55.5) |  |
| 75-79 | 2,108 (26.0) | 2,914 (27.9) |  |
| 80-84 | 901 (11.1) | 1,295 (12.4) |  |
| >85 | 297 (3.7) | 435 (4.2) |  |
| Current smoker (%) | 333 (4.1) | 323 (3.1) | <0.001 |
| Systolic blood pressure, mmHg (mean (SD)) | 141.26 (15.85) | 137.86 (16.81) | <0.001 |
| BMI, kg/m² (mean (SD)) | 27.93 (3.89) | 28.09 (5.18) | 0.024 |
| Haemoglobin, g/dL (mean (SD)) | 14.87 (1.11) | 13.64 (0.99) | <0.001 |
| HDL-c, mmol/L (mean (SD)) | 1.40 (0.39) | 1.73 (0.46) | <0.001 |
| Non-HDL-c, mmol/L (mean (SD)) | 3.62 (0.90) | 3.71 (0.97) | <0.001 |
| Diabetes (%) | 990 (12.2) | 910 (8.7) | <0.001 |
| Serum creatinine, mg/dL (mean (SD)) | 1.02 (0.21) | 0.81 (0.18) | <0.001 |
| Family history of MI (%) | 171 (2.1) | 302 (2.9) | 0.001 |
| IRSAD score (mean (SD)) | 1,003.78 (69.35) | 1,002.68 (68.78) | 0.309 |
| Intake of antihypertensive agents (%) | 3,967 (48.8) | 5,745 (55.1) | <0.001 |

Abbreviations: US = United States; SD = standard deviation; BMI = body-mass-index; HDL-c = high-density lipoprotein cholesterol; MI = myocardial infarction; IRSAD = Index of Relative Socio-economic Advantage and Disadvantage.

Table S3: Outcome events in the derivation and validation datasets

|  | Overall cohort | Males | Females |
| --- | --- | --- | --- |
| **Derivation dataset (complete cases)** | | | |
| N | 17,742 | 7,741 | 10,001 |
| MACE | 594 (3.4) | 350 (4.5) | 244 (2.4) |
| Time to first MACE | 1,669 (1,094-2,244) | 1,645 (1,027-2,263) | 1,693 (1,145-2,241) |
| **Validation dataset** | | | |
| N | 25,138 | 11,884 | 13,254 |
| MACE | 2,340 (9.3) | 1,355 (11.4) | 985 (7.4) |
| Time to first MACE | 1,286 (655-1,893) | 1,249 (623-1,867) | 1,328 (720-1,929) |

Abbreviations: CHD = coronary heart disease; MI = myocardial infarction, MACE = major cardiovascular event. All times are median (IQR) in days.

Table S4: Univariable model for prediction of incident MACE including all predictors in the derivation dataset

|  | Hazard ratio | 95% CI | p-value |
| --- | --- | --- | --- |
| Age per year | 1.08 | (1.10; 1.10) | <0.001 |
| Female Sex (yes/no) | 0.52 | (0.44; 0.61) | <0.001 |
| Current Smoking (yes/no) | 1.77 | (1.30; 2.50) | <0.001 |
| Systolic blood pressure per 10 mmHg | 1.12 | (1.10; 1.20) | <0.001 |
| Diastolic blood pressure per 10 mmHg | 1.03 | (0.96; 1.10) | 0.41 |
| BMI per kg/m^2^ | 1.01 | (0.99; 1.00) | 0.25 |
| Haemoglobin per g/dL | 1.11 | (1.00; 1.20) | 0.0023 |
| Non-HDL-c per mmol/L | 0.52 | (0.43; 0.64) | <0.001 |
| HDL-c per mmol/L | 1.24 | (1.10; 1.30) | <0.001 |
| Serum creatinine per 0.1 mg/dL | 1.13 | (1.10; 1.20) | <0.001 |
| Diabetes (yes/no) | 1.29 | (1.00; 1.60) | 0.039 |
| Family history of MI (yes/no) | 1.19 | (0.74; 1.90) | 0.47 |
| Intake of antihypertensive agents (yes/no) | 1.34 | (1.10; 1.60) | <0.001 |
| IRSAD score per unit | 1.00 | (1.00; 1.00) | 0.15 |

Abbreviations: CI = confidence interval; BMI = body-mass-index; HDL-c = high-density lipoprotein cholesterol; MI = myocardial infarction; IRSAD = Index of Relative Socio-economic Advantage and Disadvantage.

Table S5: Multivariable model for prediction of incident MACE including all predictors in the derivation dataset

|  | Hazard ratio | 95% CI | p-value |
| --- | --- | --- | --- |
| Age per year | 1.08 | (1.07; 1.10) | <0.001 |
| Female Sex (yes/no) | 0.59 | (0.48; 0.74) | <0.001 |
| Current Smoking (yes/no) | 1.91 | (1.34; 2.73) | <0.001 |
| Systolic blood pressure per 10 mmHg | 1.07 | (1.01; 1.14) | 0.021 |
| Diastolic blood pressure per 10 mmHg | 0.98 | (0.89; 1.09) | 0.75 |
| BMI per kg/m^2^ | 1.00 | (0.98; 1.02) | 0.67 |
| Haemoglobin per g/dL | 0.98 | (0.91; 1.06) | 0.64 |
| Non-HDL-c per mmol/L | 1.29 | (1.19; 1.41) | <0.001 |
| HDL-c per mmol/L | 0.77 | (0.62; 0.96) | 0.021 |
| Serum creatinine per 0.1 mg/dL | 1.05 | (1.02; 1.09) | 0.005 |
| Diabetes (yes/no) | 1.19 | (0.92; 1.54) | 0.18 |
| Family history of MI (yes/no) | 1.28 | (0.80; 2.05) | 0.30 |
| Intake of antihypertensive agents (yes/no) | 1.29 | (1.08; 1.53) | 0.004 |

This model is based on 17,649 individuals. Abbreviations: CI = confidence interval; BMI = body-mass-index; HDL-c = high-density lipoprotein cholesterol; MI = myocardial infarction.

Table S6: Area under the curve to discriminate between incident discrimination between events (MACE) and no events

|  |  | AUC (95% CI) |
| --- | --- | --- |
| SCORE2-OP model | Derivation dataset | 66.31 (64.00; 68.61) |
| Newly derived model | Derivation dataset | 68.11 (65.86; 70.35) |
|  | Derivation dataset (bias-corrected) | 67.52 |
|  | Validation dataset | 64.16 (62.77; 65.55) |

Abbreviations: AUC = Area under the curve; CI = confidence interval.

Table S7: Baseline characteristics of the validation dataset

|  | Overall cohort | Males | Females |
| --- | --- | --- | --- |
| N total | 25,138 | 11,884 | 13,254 |
| Age (mean (SD)) | 73.3 (2.62) | 73.2 (2.61) | 73.3 (2.62) |
| Age categories (%) |  |  |  |
| - 70-74 | 17,980 (72) | 8,614 (73) | 9,366 (71) |
| - 75-79 | 7,158 (29) | 3,270 (28) | 3,888 (29) |
| Female sex (%) | 13,254 (53) | - | - |
| Current smoker (%) | 1,313 (5) | 655 (6) | 658 (5) |
| Systolic Blood pressure, mmHg (mean (SD)) | 136 (14.9) | 135 (14.6) | 137 (15.2) |
| HDL-c, mmol/L (mean (SD)) | 1.5 (0.45) | 1.3 (0.38) | 1.7 (0.46) |
| Non-HDL-c, mmol/L (mean (SD)) | 3.6 (0.99) | 3.5 (0.94) | 3.8 (1.00) |
| Diabetes (%) | 3,329 (13) | 1,839 (16) | 1,490 (11) |
| Serum creatinine, mg/dL (mean (SD)) | 0.91 (0.213) | 1.02 (0.206) | 0.81 (0.168) |
| Intake of antihypertensive agents (%) | 14,106 (56) | 6,558 (55) | 7,548 (57) |

Abbreviations: SD = standard deviation; HDL-c = high-density lipoprotein cholesterol.

Table S8: Multivariable model for prediction of incident MACE including all predictors, sensitivity analyses including IRSAD score in the derivation dataset

|  | Hazard ratio | 95% CI | p-value |
| --- | --- | --- | --- |
| Age per year | 1.09 | (1.07; 1.11) | <0.001 |
| Female Sex (yes/no) | 0.61 | (0.48; 0.77) | <0.001 |
| Current Smoking (yes/no) | 1.78 | (1.20; 2.63) | 0.004 |
| Systolic blood pressure per 10 mmHg | 1.06 | (0.99; 1.13) | 0.08 |
| Diastolic blood pressure per 10 mmHg | 0.96 | (0.87; 1.07) | 0.47 |
| BMI per kg/m^2^ | 1.01 | (0.99; 1.03) | 0.35 |
| Haemoglobin per g/dL | 1.01 | (0.93; 1.09) | 0.89 |
| Non-HDL-c per mmol/L | 1.28 | (1.17; 1.40) | <0.001 |
| HDL-c per mmol/L | 0.73 | (0.58; 0.93) | 0.010 |
| Serum creatinine per 0.1 mg/dL | 1.05 | (1.02; 1.09) | 0.005 |
| Diabetes (yes/no) | 1.07 | (0.80; 1.43) | 0.64 |
| Family history of MI (yes/no) | 1.23 | (0.73; 2.05) | 0.44 |
| IRSAD score per unit | 1.00 | (1.00; 1.00) | 0.63 |
| Intake of antihypertensive agents (yes/no) | 1.26 | (1.05; 1.52) | 0.012 |

This model is based on 15,773 individuals. Abbreviations: CI = confidence interval; BMI = body-mass-index; HDL-c = high-density lipoprotein cholesterol; MI = myocardial infarction; IRSAD = Index of Relative Socio-economic Advantage and Disadvantage.

Table S9: Weighting of variable selection using least absolute shrinkage and selection operator as sensitivity analysis including IRSAD as predictor in the derivation dataset

| Predictor | % |
| --- | --- |
| Age | 100.0% |
| Sex | 97.5% |
| Non-HDL-c | 88.0% |
| HDL-c | 85.5% |
| Serum creatinine | 71.5% |
| Current smoking | 25.5% |
| Systolic blood pressure | 21.5% |
| Intake of antihypertensive agents | 11.0% |
| BMI | 4.5% |
| Haemoglobin | 4.0% |
| IRSAD score | 3.5% |
| Diabetes | 1.5% |
| Family history of MI | 1.0% |

Abbreviations: BMI = body-mass-index; HDL-c = high-density lipoprotein cholesterol; MI = myocardial infarction; IRSAD = Index of Relative Socio-economic Advantage and Disadvantage.

Table S10: Explorative analyses of the multivariable regression model in subgroups by sex in the derivation dataset

|  | Males | | | Females | | |
| --- | --- | --- | --- | --- | --- | --- |
|  | **Hazard ratio** | **95% CI** | **p-value** | **Hazard ratio** | **95% CI** | **p-value** |
| Age per year | 1.07 | (1.05; 1.09) | <0.001 | 1.11 | (1.08; 1.13) | <0.001 |
| Current smoking (yes/no) | 1.73 | (1.10; 2.71) | 0.018 | 2.39 | (1.39; 4.10) | 0.002 |
| Systolic blood pressure per 10 mmHg | 1.08 | (1.01; 1.15) | 0.029 | 1.04 | (0.97; 1.12) | 0.25 |
| Non-HDL-c per mmol/L | 1.32 | (1.17; 1.48) | <0.001 | 1.25 | (1.10; 1.42) | <0.001 |
| HDL-c per mmol/L | 0.75 | (0.55; 1.02) | 0.06 | 0.78 | (0.58; 1.06) | 0.12 |
| Diabetes (yes/no) | 1.32 | (0.96; 1.80) | 0.08 | 1.03 | (0.66; 1.62) | 0.90 |
| Serum creatinine per 0.1 mg/dL | 1.06 | (1.02; 1.11) | 0.007 | 1.03 | (0.97; 1.10) | 0.34 |
| Intake of antihypertensive agents (yes/no) | 1.24 | (1.00; 1.54) | 0.05 | 1.43 | (1.09; 1.88) | 0.010 |

This model is based on 7,741 individuals and 350 events for males and 10,001 individuals and 244 events for females. Abbreviations: CI = confidence interval; HDL-c = high-density lipoprotein cholesterol.

Table S11: Baseline characteristics of the diabetic derivation population

| N | 1,900 |
| --- | --- |
| Australian participants (%) | 1,644 (86.5) |
| US participants (%) | 256 (13.5) |
| Age (mean (SD)) | 75.59 (4.46) |
| Age categories (%) |  |
| 70-74 | 1,037 (54.6) |
| 75-79 | 543 (28.6) |
| 80-84 | 237 (12.5) |
| >85 | 83 (4.4) |
| Female sex (%) | 910 (47.9) |
| Current smoker (%) | 80 (4.2) |
| Systolic blood pressure, mmHg (mean (SD)) | 140.61 (15.97) |
| BMI, kg/m² (mean (SD)) | 30.30 (4.91) |
| Haemoglobin, g/dL (mean (SD)) | 14.13 (1.31) |
| HDL-c, mmol/L (mean (SD)) | 1.38 (0.42) |
| Non-HDL-c, mmol/L (mean (SD)) | 3.18 (0.96) |
| Glucose, mg/dL (mean (SD)) | 131.16 (34.92) |
| Serum creatinine, mg/dL (mean (SD)) | 0.95 (0.25) |
| Family history of MI (%) | 41 (2.2) |
| Intake of statin (%) | 1,126 (59.3) |
| Intake of ACEI/ARB (%) | 1,236 (65.1) |
| Intake of antihypertensive agents (%) | 1,415 (74.5) |
| Intake of antidiabetic agents (%) | 1,055 (55.5) |

Abbreviations: US = United States; SD = standard deviation; BMI = body-mass-index; HDL-c = high-density lipoprotein cholesterol; MI = myocardial infarction; ACEI = angiotensin-converting-enzyme inhibitor; ARB = angiotensin-receptor blocker.

**Supplementary Figures**

Figure S1: Correlation matrix of continuous baseline variables in the derivation dataset


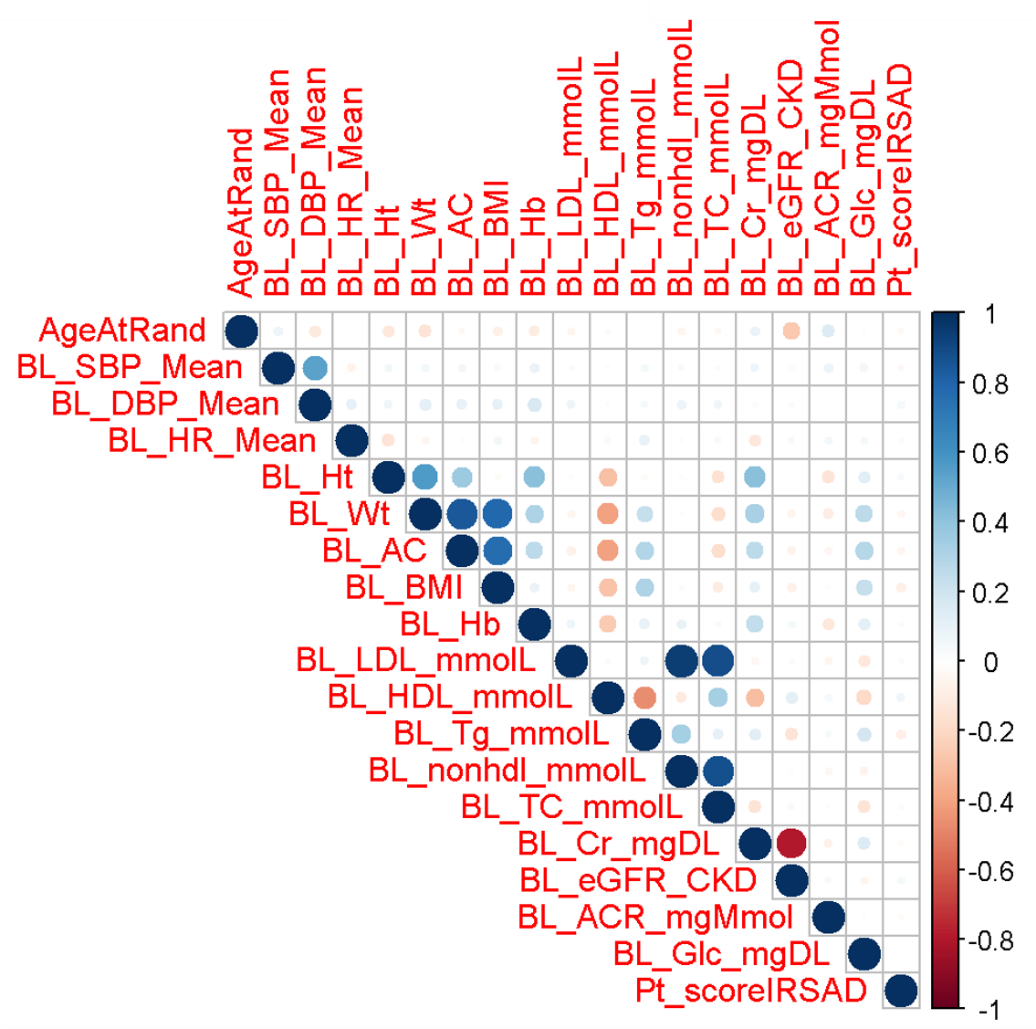


This plot depicts the correlation coeffects of all continuous variables in the dataset.

Abbreviations: BL = baseline; SBP = systolic blood pressure, DBP = diastolic blood pressure; HR = heart rate; Ht = height; Wt = weight; BMI = body-mass-index; Hb = haemoglobin; LDL = low-density lipoprotein; HDL = high-density lipoprotein; Tg = triglycerides; nonHDL = non-high-density lipoprotein; TC = total cholesterol; Cr = creatinine; eGFR = estimated glomerular filtration rate; ACR = albumin-creatinine-ratio; Glc = glucose; IRSAD = Index of Relative Socio-economic Advantage and Disadvantage.

Figure S2: Forest plots of univariable models for prediction of incident MACE in the derivation dataset

Abbreviations: BMI = body-mass-index; HDL-c = high-density lipoprotein cholesterol; MI = myocardial infarction; IRSAD = Index of Relative Socio-economic Advantage and Disadvantage.

Figure S3: Forest plots of the final model for the overall population, for males, and for females in the derivation dataset


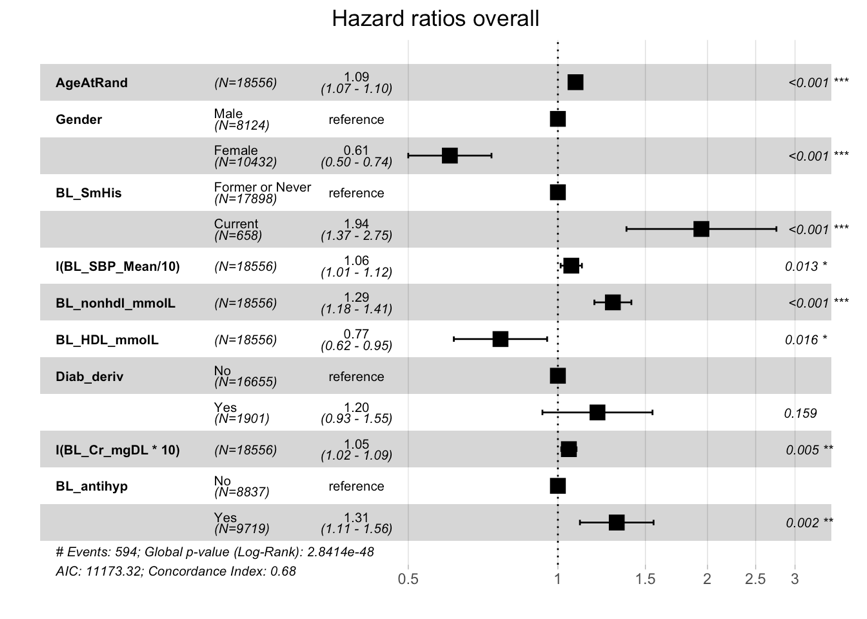


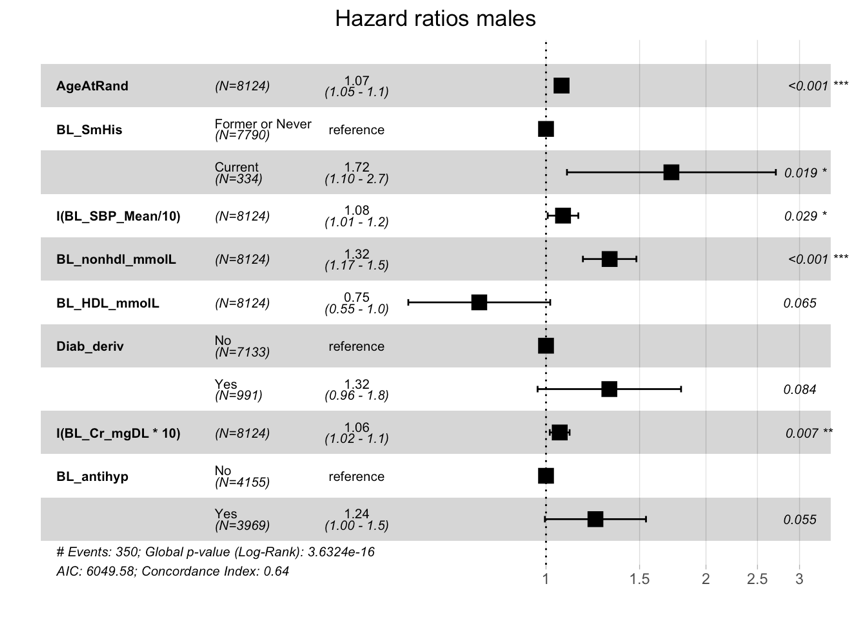


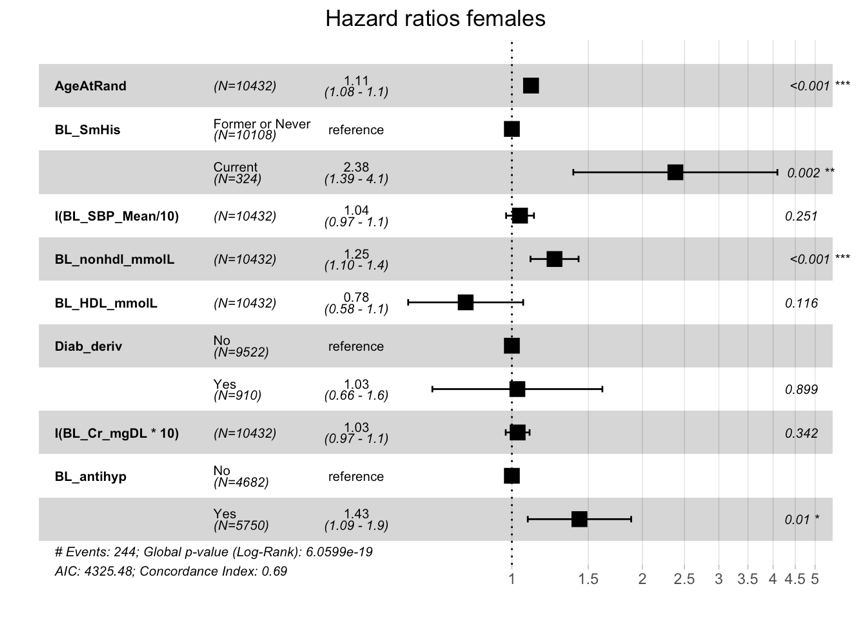


The forest plots display the hazard ratios for each variable predicting incident MACE.

Figure S4: Calibration plot of the final prediction model for males and females in the derivation dataset


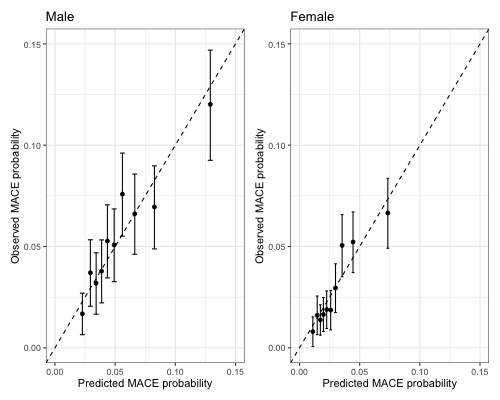


The black dots compare the observed and the predicted probability of MACE together with the 95% confidence intervals.
